# Effects of currently prescribed LDL-C-lowering drugs on PCSK9 and implications for the next generation of LDL-C-lowering agents

**DOI:** 10.1186/1476-511X-10-38

**Published:** 2011-02-28

**Authors:** Robert J Konrad, Jason S Troutt, Guoqing Cao

**Affiliations:** 1 Lilly Research Laboratories, Eli Lilly and Company, Indianapolis, IN 46285. USA

## Abstract

**Background:**

During the past decade, proprotein convertase subtilisin kexin type 9 (PCSK9) has been identified as a key regulator of serum LDL-cholesterol (LDL-C) levels. PCSK9 is secreted by the liver into the plasma and binds the hepatic LDL receptor, causing its subsequent degradation. In humans, gain-of-function mutations in PCSK9 cause a form of familial hypercholesterolemia that manifests with dramatically increased serum levels of LDL-C, while loss-of-function mutations in PCSK9 are associated with significantly decreased LDL-C and cardiovascular risk.

**Results:**

Initial studies in animals and cultured cells demonstrated that statins increased PCSK9 mRNA expression, resulting in many research groups exploring the effect of statins on PCSK9 levels in humans. We first reported that statins increased human PCSK9 circulating protein levels. Additional researchers subsequently confirmed these observations, further prompting many laboratories including our own to examine the effect of other lipid lowering medications on PCSK9 levels. Our observation that fenofibrate (200 mg/day) significantly increased PCSK9 levels was confirmed by another laboratory, and an additional group demonstrated that ezetimibe also increased PCSK9 levels.

**Conclusions:**

It has become clear that the major classes of commonly prescribed lipid-lowering medications increase serum PCSK9 levels. These observations almost certainly explain why these agents are not more effective in lowering LDL-C and suggest that efforts should be made toward the development of new LDL-C lowering medications that either do not increase circulating PCSK9 levels or work through decreasing or inhibiting PCSK9.

## Discovery of PCSK9, mechanism of action, and genetic findings

The discovery of proprotein convertase subtilisin kexin type 9 (PCSK9) and the subsequent observations that genetic mutations in PCSK9 can drastically affect low density lipoprotein cholesterol (LDL-C) levels and the risk for cardiovascular disease ushered in a new era of understanding about the regulation of circulating LDL-C [1-6]. PCSK9 was first described by Seidah and co-workers in 2003 as a protein important in liver regeneration and neuronal differentiation [7]. These researchers originally named the protein (which was the ninth described member of the proprotein subtilisin kexin family of proteins) neuronal apoptosis regulated convertase-1 (NARC-1).

Later that year, Abifadel and colleagues reported the discovery of 2 mutations in PCSK9 that resulted in familial hypercholesterolemia (FH), a genetic disease characterized by greatly increased levels of LDL-C and early onset of atherosclerosis and cardiovascular events [8]. Prior to that report, FH had been associated with mutations in either the low density lipoprotein receptor (LDLR) or apolipoprotein B (ApoB). Mutations in these 2 genes however, could not account for all cases of FH, and the observation that PCSK9 mutations could result in FH established PCSK9 as third important locus of FH [8].

**Figure 1:**
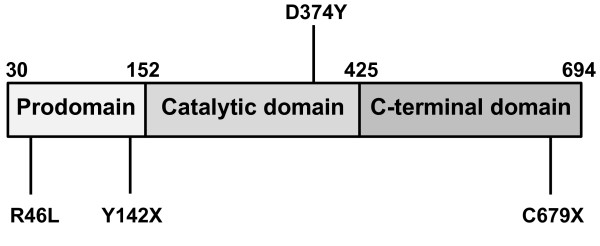
**PCSK9 protein structure and selected mutations**. PCSK9 protein includes a pro-domain (Pro) that is self-cleaved during secretion and remains tightly associated, a catalytic domain, and a C-terminal domain. Activating mutations such as D374Y cause increased affinity for LDLR. Inactivating mutations such as Y142X and C679X block self-cleavage and secretion of the protein. The R46L mutation results in decreased circulating levels of PCSK9 protein, although the mechanism is not completely understood.

In 2004, Maxwell and colleagues reported that overexpression of PCSK9 in mice resulted in a phenotype that was very similar to that of an LDLR knockout model, with large increases in circulating non high density lipoprotein cholesterol (HDL-C), and a virtual absence of measureable LDLR protein [9]. In addition, these researchers observed that PCSK9 overexpression had little effect on LDLR mRNA levels suggesting that PCSK9 was acting at the protein level through degradation of the hepatic LDLR [9]. These interesting observations were followed by a number of reports over the next few years further elucidating the mechanism by which PCSK9 decreases LDLR levels. It was gradually recognized that PCSK9 was a protease secreted by the liver into the plasma, which was then able to bind to and cause the degradation of hepatic LDLR [10-19]. The mechanism by which PCSK9 degrades LDLR has turned out to be more complex than originally anticipated. As Figure 1 shows, PCSK9 protein contains a pro-domain that is self-cleaved during secretion and remains tightly associated with the protein, as well as catalytic and C-terminal domains. Recent studies suggest that after self-cleavage and secretion, PCSK9 does not have to be further enzymatically active to cause degradation of the LDLR [20-22]. Instead, PCSK9 has been shown to bind to the LDLR and subsequently target it for lysosomal destruction within the hepatocyte [20-22]. This concept of how PCSK9 acts to decrease hepatic LDLR levels has been further supported by findings that disruption of the binding of PCSK9 to the LDLR using anti-PCSK9 antibody results in preserved LDLR and decreased LDL-C [23-26].

At the same time that the biology of PCSK9 was being increasingly understood, additional different activating PCSK9 mutations causing FH were being reported in humans. It was increasingly recognized that these patients were presenting with gain-of-function mutations of PCSK9, causing LDLR protein levels to be markedly decreased with resulting FH and accompanying increased cardiovascular risk [27-29]. Interest in PCSK9 then dramatically accelerated in 2006 with the report by Hobbs and co-workers that approximately 3% of African-Americans were heterozygous for loss-of-function mutations in PCSK9, including mutations such as Y142X and C679X which prevent the self-cleavage and secretion of the protein, and that these subjects had significantly decreased levels of serum LDL-C and approximately 80-90% reduced cardiovascular risk [30]. Later that same year, Hobbs and co-workers described a compound heterozygote for PCSK9 loss-of-function mutations [31]. Remarkably, the subject who was a healthy 32 year-old female, had a serum LDL-C level of 14 mg/dl [31]. More recently, a second subject, a 49 year-old male, heterozygous for 2 monoallelic dominant negative PCSK9 mutations has also been described and had a LDL-C of 16 mg/dl [32].

Shortly after the loss-of-function findings in African-Americans were described, it was also reported that 3% of Caucasian subjects, while not possessing the complete loss of function mutations observed in African-Americans, did harbor mutations in PCSK9 that resulted in decreased serum cholesterol levels [33]. Among these mutations, one of the most prominent was R46L, which conferred a 10-20% reduction in circulating LDL-C and a 50% reduced risk of cardiovascular disease [33]. It was not intuitively obvious why the R46L mutation should do this. Only when the laboratories of Humphries and co-workers and of Hobbs and co-workers reported that the R46L mutation resulted in significantly decreased levels of circulating PCSK9 did it become apparent that the mutation was somehow lowering the amount of PCSK9 protein in the serum, and that this reduction in serum PCSK9 was likely responsible for the beneficial effects on LDL-C and cardiovascular risk [34,35].

In light of all of the findings in humans showing that PCSK9 mutations that decrease PCSK9 levels result in decreased serum LDL-C and cardiovascular risk, there has been tremendous increased interest in understanding the normal role of PCSK9. In 2006, Costet and colleagues reported that PCSK9 mRNA expression was decreased 73% in mice after fasting for a 24-hour period and that PCSK9 mRNA expression levels returned to normal following re-feeding with carbohydrate [36]. In the same study, it was also shown that insulin increased PCSK9 mRNA expression levels, suggesting that PCSK9 expression may be regulated by nutritional status via circulating insulin levels [36].

Recently, Horton and colleagues examined the relationship between lathosterol, a marker of hepatic cholesterol biosynthesis and circulating PCSK9 levels in humans [37]. This group observed that lathosterol and PCSK9 levels were highly correlated with each other and declined together during the fasting state. Around the same time, Rudling and colleagues conducted extensive studies on the effect of prolonged fasting as well as the administration of the bile acid binding resin cholestyramine on circulating PCSK9 and lathosterol and observed that PCSK9 levels and lathosterol levels move in virtual lockstep with each other and are both dramatically decreased by fasting, whereas LDL cholesterol was little changed [38]. Together, these results would seem to indicate that PCSK9 may represent a mechanism that has evolved for fine-tuning plasma LDL-C levels, although this idea will have to be further probed. In any event, all of the findings described above certainly established PCSK9 as a major player in the regulation of LDL-C levels.

## Statins, PCSK9, and the rule of 6%

Statins represent the most commonly prescribed class of LDL-C lowering medications. Statins work through inhibiting the enzyme 3-Hydroxy-3-methyl-glutaryl-CoA reductase (HMG-CoA reductase) which represents the rate limiting step in cholesterol biosynthesis [39]. By decreasing hepatic intracellular levels of cholesterol, statins increase the activity/nuclear translocation of the transcription factor sterol regulatory element-binding protein-2 (SREBP-2), which activates the LDLR gene [40]. Increased expression of LDLR on the surface of hepatocytes results in increased LDL binding and catabolism which in turn decreases circulating LDL-C levels by 30-40% or even more [40].

In 2003, Maxwell and co-workers first identified PCSK9 as a novel target gene of SREBP and demonstrated that hepatic PCSK9 mRNA levels were significantly increased in SREBP-1a and SREBP-2 transgenic mice [41]. In 2004, Dubuc and colleagues reported in a study using HEPG2 cells and primary hepatocytes that statins upregulate the expression of PCSK9 mRNA [42]. The induction of PCSK9 mRNA was described as being highly dose dependent and was reversed by mevalonate [42]. The authors also observed that human, mouse, and rat PCSK9 promoters contain 2 typical conserved motifs for cholesterol regulation, a sterol regulatory element (SRE) and a Sp1 site. They concluded that PCSK9 regulation is typical of that of the genes implicated in lipoprotein metabolism (such as LDLR) and that PCSK9 was likely a target of SREBP-2 [42]. The following year, Horton and co-workers demonstrated that PCSK9 knockout mice exhibited an exaggerated response to statin administration as demonstrated by increases in hepatic LDLR and increased LDL-C clearance from their plasma [43]. Together with additional results generated in other laboratories [44-46], these data further supported the notion that statins increase the activity/nuclear translocation of SREBP-2, a transcription factor that activates both the LDLR and PCSK9 genes [41-46]. These data gave the first indication *in vivo *that a PCSK9 inhibitor might be able to reduce plasma cholesterol beyond what was achievable with statins alone [41-46].

These observations led a number of laboratories, including our own, to examine the effects of statins on human PCSK9 levels. In order to do this, there were 2 requirements. The first was a reliable immunoassay for measuring human serum PCSK9 levels. The second was a set of samples to test from patients who had blood drawn at various time points prior to and after the initiation of statin therapy. With regard to the development of a reliable immunoassay, our group had begun working on this goal in early 2006, and by 2007 had established a robust, dual-monoclonal sandwich enzyme linked immunosorbent assay (ELISA) that we were able to use to show that human serum PCSK9 levels were highly correlated with LDL-C levels [47]. Later that same year, we were able to obtain a set of baseline and endpoint samples from patients treated with placebo, 10 mg/day atorvastatin, or 40 mg/day atorvastatin for 12 weeks. Using our sandwich ELISA, we were able to demonstrate that atorvastatin (40 mg/day) increased serum PCSK9 levels by 34% compared to placebo at 12 weeks, while lowering LDL-C by 42% [48]. In contrast, placebo treatment in the same study had no significant effect on either LDL-C levels or serum PCSK9 levels [48].

The fact that atorvastatin significantly increased PCSK9 levels provided evidence that statins increase the activity/nuclear translocation of sterol regulatory element-binding protein-2 (SREBP-2) to such an extent that increased levels of PCSK9 protein were present in the serum. We concluded this was particularly important since the ability to detect any increase in secreted PCSK9 protein could have been negated by the increased hepatic LDLR that would act to bind PCSK9 and remove it from the circulation. Therefore, we hypothesized that atorvastatin treatment must have increased expression and secretion of PCSK9 protein to such an extent that circulating PCSK9 levels exceeded LDLR binding, resulting in increased circulating levels of PCSK9 protein being detected in serum.

Our initial observation that statin treatment in humans caused an increase in serum PCSK9 levels was soon confirmed by other groups, further suggesting the importance of statin-induced increases in serum PCSK9 levels [34,49,50]. As interesting as these observations were, they were somewhat limited in that the baseline and endpoint analysis of samples did not allow for the detailed time course of statin-induced PCSK9 increases to be described. It was thus not clear at the time how quickly the effect of statins on PCSK9 levels occurred or whether the effect was sustained over time. As a result, we conducted an additional larger study examining the detailed time course of the effect of 80 mg/day atorvastatin on human serum PCSK9 levels in which we measured serum PCSK9 and lipid levels during a 2-week lead-in baseline period and every 4 weeks thereafter for 16 weeks. We observed in this subsequent study that atorvastatin (80 mg/day) caused a rapid 47% increase in serum PCSK9 after 4 weeks of treatment that was completely sustained throughout 16 weeks of dosing [51]. Interestingly, we also observed that while PCSK9 levels were highly correlated with LDL-C at baseline, atorvastatin (80 mg) completely abolished this correlation [51].

In this study, we also compared baseline PCSK9 levels to atorvastatin-induced changes in LDL-C (from baseline to endpoint) to determine if baseline PCSK9 levels might predict whom would respond most robustly to atorvastatin treatment. We hypothesized that subjects who had the highest PCSK9 levels might also have the largest atorvastatin-induced LDL-C lowering effect. Consistent with this hypothesis, there was a modest relationship between baseline PCSK9 levels and changes in LDL-C, with relatively higher baseline PCSK9 levels tending to be associated with numerically greater decreases in LDL-C [51]. This correlation, however, did not achieve statistical significance [51].

These data, in conjunction with those of Costet and colleagues who recently showed that atorvastatin 10 mg/day increased PCSK9 levels by only 14% in patients after 6 weeks of therapy [52], suggested a clear dose response effect for atorvastatin on PCSK9 levels, with increasing doses of atorvastatin causing larger and larger percentage increases in circulating PCSK9 levels. Available data regarding the effect of atorvastatin on human serum PCSK9 levels in controlled clinical trials where endpoint PCSK9 levels could be compared to baseline levels is summarized in Table 1, which shows that increasing doses of atorvastatin result in dose-dependent increases in circulating PCSK9 protein. Consistent with this pattern, Lakoski and co-workers recently demonstrated in a large trial that at a low dose of simvastatin (10 mg/day), there was no significant increase in median circulating PCSK9 levels [53].

**Table 1:** Effect of increasing doses of atorvastatin on serum PCSK9 levels

Atorvastatin Dose (mg/day)	Duration of treatment (weeks)	Increase in serum PCSK9 levels (%)	Author and study (reference)
10	6	14	Costet et al. [52]

40	12	34	Careskey et al. [48]

80	16	47	Welder et al. [51]

These results summarize the available data regarding the effect of atorvastatin on human serum PCSK9 levels in controlled clinical trials in which endpoint PCSK9 levels could be compared to baseline levels. Treatment with increasing doses of atorvastatin resulted in dose-dependent increases in the levels of circulating PCSK9 protein.

**Figure 2:**
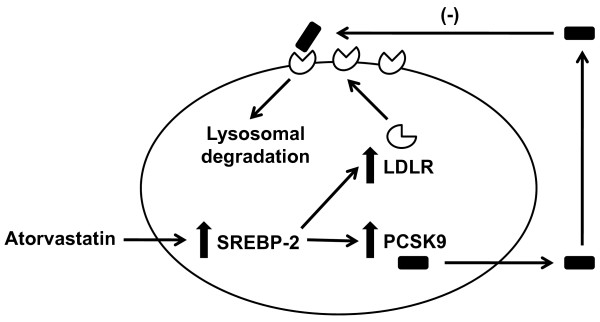
**Effect of atorvastatin on hepatocyte LDLR and PCSK9**. Atorvastatin increases the activity/nuclear translocation of sterol regulatory element-binding protein-2 (SREBP-2), which is a transcription factor that activates both the LDLR and PCSK9 genes. This results in increased expression and secretion of PCSK9 protein, which binds the LDLR and targets it for lysosomal degradation. This likely prevents atorvastatin from causing as much increased LDLR protein from being present on the hepatocyte plasma membrane as might otherwise be expected.

As Figure 2 demonstrates, these data suggest that the explanation for why increasing doses of statins fail to achieve proportional LDL-C lowering is that statins increase PCSK9 protein levels in a dose-dependent fashion, and that the increased PCSK9 levels largely negate further statin-induced increases in hepatic LDLR levels. For some time, it has been known that statins follow a rule of 6% in that whatever LDL-C reduction is achieved at a starting dose of a given statin is only improved upon an additional approximate 6% with each doubling of the dose. This important observation of non-dose dependent response for commonly prescribed statins has led to much speculation about why statins should affect LDL-C levels in such a manner. Our own data, combined with data that other laboratories have generated, suggest that statin-induced increases in PCSK9 protein levels account for the less than expected, incremental LDL-C lowering observed when the starting dose of a statin is subsequently increased.

Besides statins, another commonly prescribed LDL-C lowering medication is ezetimibe, which acts by blocking uptake of dietary cholesterol from the gut by binding the cholesterol transport protein Niemann-Pick C1-Like 1 (NPC1L1) [40,54-56]. By decreasing the amount of cholesterol supplied to the liver through intestinal uptake, ezetimibe decreases hepatic cholesterol levels, leading to increased hepatic LDLR expression, which results in increased LDL uptake from the plasma and thus decreased circulating LDL-C levels [40,54-56]. Ezetimibe lowers LDL-C by approximately 15-20%, which is a smaller decrease than that achievable with statins, but its effect appears to be additive to that of statins [40,54-56].

With regard to the effect of ezetimibe on PCSK9 levels, there have been fewer studies than for statins. A recent reports by Davignon and colleagues indicated that when added onto statin therapy, ezetimibe further increased PCSK9 levels beyond the increases observed with statins alone [57]. In this study, the researchers observed that patients treated with statins alone had an approximately 45% increase in circulating PCSK9 levels, while those treated with a combination of statins and ezetimibe exhibited an approximately 77% increase in PCSK9 protein levels [57]. At the present time, the mechanism by which ezetimibe causes a further increase in PCSK9 levels compared to statin treatment alone is unclear, but is likely associated with reduced intestinal cholesterol absorption and thus reduced hepatic cholesterol to feedback regulate SREBP2 [40]. As a result of these interesting findings, further study in this area will clearly be warranted. Of particular importance will be to determine the effect of ezetimibe alone on serum PCSK9 levels and how it is modulated with the addition of increasing doses of statins.

## Fibrates and their effects on PCSK9 levels

Fibrates represent a class of medications that activate the transcription factor peroxisome proliferator-activated receptor α (PPARα) [58,59]. Activation of this receptor by fibrates alters the transcription of multiple target genes which play a key role in lipid metabolism [58,59]. In particular fibrates have been shown to reduce plasma levels of triglycerides by approximately 30%-50% and have been demonstrated to increase levels of HDL-C by 5%-15% [58,59]. The effects on LDL-C are less consistent. In some cases, fibrates may reduce LDL-C by as much as 15%-20% although the effect can be variable, depending on the patient population being studied and the type of hyperlipidemia present [58,59].

Previous data showing that statins increase PCSK9 levels caused many researchers including our own group to wonder about the effects of fibrates on PCSK9 levels. To address this question, we studied the effect of a commonly prescribed fibrate, fenofibrate, on circulating PCSK9 protein levels in patients treated with either fenofibrate (200 mg/day) or placebo for 12 weeks. We observed that fenofibrate significantly increased circulating PCSK9 levels by 25% compared to baseline, while placebo treatment had no effect on PCSK9 levels [60]. Interestingly, fenofibrate-induced increases in serum PCSK9 levels were highly correlated with fenofibrate-induced changes in LDL-C levels. Similar to what was observed with statins, these results suggested an explanation for why fibrates might not achieve as much LDL-C lowering as might otherwise be expected [60].

The mechanism by which fenofibrate increased PCSK9 levels in this study was unclear. Fenofibrate belongs to a class of drugs called PPAR-α agonists, which affect lipid levels by altering transcription of a number of different genes involved in lipoprotein and fatty acid metabolism [61]. As a PPAR-α agonist, fenofibrate reduces hepatic triglyceride synthesis and increases the breakdown of triglyceride rich lipoproteins, while also enhancing cholesterol efflux from the liver [62]. It is possible that these effects on cholesterol and lipoprotein metabolism may work indirectly within the hepatocyte to decrease intracellular cholesterol levels, thus leading to increased PCSK9 expression and secretion via a mechanism similar to that of statins. In light of the multiple pleiotropic effects of fenofibrate [63], however, it is also possible that fenofibrate may exert a direct effect within hepatocytes to stimulate increased PCSK9 synthesis and secretion independent of SREBP-2.

Making matters somewhat more complex, there have been conflicting observations concerning the effect of fibrates on hepatocyte PCSK9 synthesis and secretion. Previously, Mayne and co-workers reported that fibrates increased human serum PCSK9 levels by 17% [64]. In this study, however, analysis of PCSK9 levels was performed using an immunoprecipitation and western blotting method, which is a semi-quantitative rather than a quantitative technique. In addition, the absolute levels of PCSK9 present in human serum as reported by this method were significantly greater than what our laboratory as well as several others have reported. Also, the group of patients analyzed in this study was relatively small and consisted of a mixture of patients on gemfibrozil and fenofibrate.

In contrast to this report, Kourimate and co-workers demonstrated that fibrate treatment resulted in reduced PCSK9 mRNA levels in hepatocytes [65]. This group also went on to measure levels of PCSK9 protein expression in hepatocytes and concluded that PCSK9 protein expression in hepatocytes was also reduced. Again, similar to the work by Mayne and co-workers, PCSK9 protein levels were measured by western blotting. Kourimate and co-workers concluded from these data that addition of a fibrate to pre-existing statin therapy would result in suppression of circulating PCSK9 levels and thus enhanced LDL-C lowering activity of the statin. On the other hand, Lambert and colleagues used an ELISA method to report that fenofibrate treatment (200 mg per day for 6 weeks) decreased plasma PCSK9 levels by 8% and that this decrease correlated with the fenofibrate-induced decreases in circulating triglycerides [66]. Similar to the observations by Mayne and colleagues, the absolute plasma levels of PCSK9 as reported by this method were significantly greater than what our laboratory and several other groups have reported. The same researchers concluded that fenofibrate-induced decreases in PCSK9 might account for the modest LDL-C reduction observed with fenofibrate [66].

The conclusions reached by these two groups were different from those that we reached with regard to the effect of fibrates on circulating PCSK9 protein levels. One possibility for this difference may have been the use of different analytical techniques than those used by our laboratory and other laboratories. This possibility seems even more likely in light of a very recent, thorough study exploring the effects of fenofibrate on PCSK9 serum levels in patients with diabetes. In their recent report using a robust and specific assay, Costet and colleagues demonstrated that 6 weeks of treatment with fenofibrate (160 mg/day) increased serum PCSK9 by 26% [52]. This increase in circulating PCSK9 levels was very similar to the increase of 25% that we observed after 12 weeks of treatment using our own assay.

Therefore, at the current time, it seems likely that fibrates share with statins the common property of increasing serum PCSK9 protein levels. Unlike the situation for statins though, in which the mechanism for the increase in PCSK9 is likely mediated through SREBP-2, it is much less understood how fibrates actually increase circulating PCSK9 protein levels. Certainly additional studies will be needed to better understand the biology of fibrate-induced PCSK9 increases.

## Future directions for LDL-lowering therapies

In light of the genetic studies in humans showing PCSK9 mutations that decrease PCSK9 levels result in decreased serum LDL-C and cardiovascular risk and the findings that the utility of both statins and fibrates may be limited due to the fact that they increase serum PCSK9 levels, there has been tremendous increased interest in developing a PCSK9 inhibitor. One approach that has been tested is to use an antisense oligonucleotide (ASO) to target PCSK9 mRNA. Graham and colleagues demonstrated that this approach reduced total cholesterol by 53% in mice fed a high-fat diet, while also causing a 2-fold increase in hepatic LDLR protein levels [14]. Similarly, Gupta and co-workers reported that an ASO lowered PCSK9 mRNA expression in mice by 60% and increased hepatic LDLR protein expression by almost 3-fold [67]. Comparable results have also been reported using a siRNA approach by Fitzgerald and colleagues, who demonstrated 60% reductions in PCSK9 mRNA and total cholesterol in mice [68]. This same group also demonstrated that such an approach was possible in a non-human primate model as well by showing that a single siRNA injection produced long-lasting reductions in both serum PCSK9 and LDL-C levels [68].

Due to the nature of PCSK9 protein and the way in which it functions by binding the LDLR, several groups have also attempted to generate neutralizing, fully humanized anti-PCSK9 monoclonal antibodies to block the interaction of PCSK9 with the LDLR at the protein level. Preliminary results so far have been encouraging. Chan and colleagues developed a monoclonal antibody that binds a PCSK9 epitope required for recognition by the LDLR. When administered to cynomolgus monkeys, one injection of this antibody decreased serum LDL-C by approximately 80%, and a decrease in LDL-C was observed over the course of 10 days [25]. In a separate, recent report, Ni and co-workers developed a monoclonal PCSK9 antibody that was neutralizing not only to wild type PCSK9 but also to gain of function mutants including D374Y [26]. When this antibody was administered to rhesus monkeys, LDL-C was decreased from 20-50% over a 2-week long period, even though the antibody had a relatively short half-life.

These observations clearly established a proof-of-concept that a neutralizing anti-PCSK9 monoclonal antibody is capable of reducing LDL-C levels in primates. One of the big remaining questions, however, relates to the durability of such a therapeutic approach in actual patients. In order to understand how feasible such an approach will ultimately be in the clinic, it is important to understand 2 key characteristics of PCSK9 - its half-life and what the absolute circulating levels are. With regard to the first issue, the half-life of PCSK9 appears to be relatively short, presumably because PCSK9 in the plasma is rapidly bound by LDLR and/or similar type receptors. The best current estimates for the circulating half-life are somewhere between 5 and 30 minutes [11,19].

**Figure 3:**
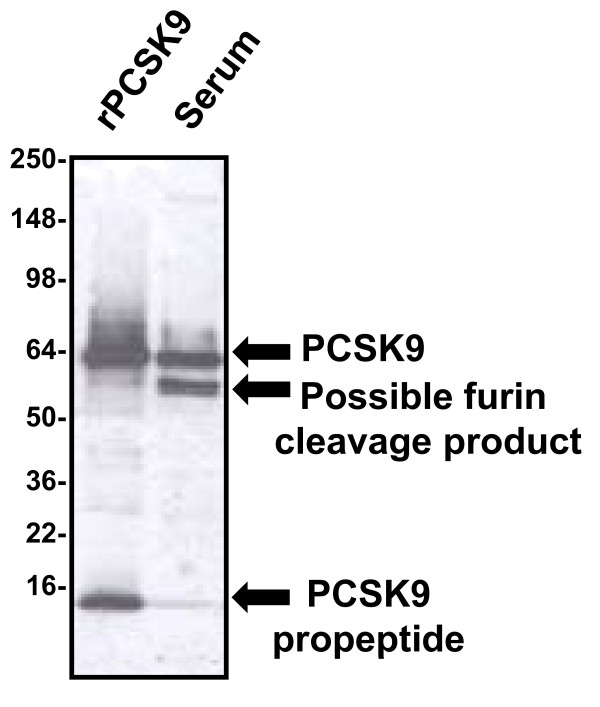
**Characterization of PCSK9 protein in human serum**. When immunoprecipitated from human serum and analyzed in our laboratory by subsequent western blotting under reducing and denaturing conditions, PCSK9 migrates as an intact band (top arrow) that co-migrates with recombinant PCSK9 protein (rPCSK9) [51,60]. In addition, a band of approximately 55 kD is also present (middle arrow), and this band had been described as representing a furin cleavage product of the intact protein [49]. The band at the very bottom of the blot represents the propeptide which remains tightly but non-covalently associated during the immunoprecipitation step, but is subsequently separated by denaturation prior to western blotting (lower arrow).

With regard to the absolute levels of circulating PCSK9 protein, there is currently less agreement. Part of the problem has been the relative difficulty of developing robust methods to measure PCSK9 in human serum. When analyzed by western blotting under reducing, denaturing conditions, intact PCSK9 protein co-migrates almost precisely with human albumin, which is present in the serum at a concentration of approximately 4 g/dl. Because of this, it is extremely difficult to accurately gauge human serum levels when performing straight western blotting procedures alone. As Figure 3 demonstrates, when suitable immunoprecipitation is performed, followed by western blotting, it becomes easier to visualize the PCSK9 band, but also apparent that there is at least one additional band which almost precisely co-migrates with human IgG heavy chain under reducing denaturing conditions. This second band has been described as a furin cleavage product of PCSK9 [49], and can be almost as intense as the intact protein itself.

Therefore, when designing a sandwich ELISA to measure PCSK9 protein, it is possible depending on the epitopes recognized by the capture and conjugate antibodies to measure either total circulating PCSK9 or just intact PCSK9. Furthermore, our laboratory has also noticed that some antibodies may have altered affinity for recombinant PCSK9 protein when it is expressed with a His tag versus having no His tag [47,48,51,60]. In addition, it has also been suggested that expressed PCSK9 protein may self-associate to form multimers [69], which might further complicate accurate measurement of PCSK9 levels in serum. To investigate this possibility, we performed experiments pairing each of our monoclonal antibodies with itself in a sandwich ELISA format. In our experience, homomultimeric proteins are able to be detected by such a configuration, but we were not able to measure any signal when using the same anti-PCSK9 monoclonal antibody as both a capture and conjugate antibody in a sandwich ELISA (unpublished data). These results suggested to us that PCSK9 may not form multimers; however, this possibility cannot be excluded. Undoubtedly, these aspects of the molecule may account at least in part for some of the difference in the absolute values of human serum PCSK9 reported using various ELISA methods.

Despite these technical challenges, however, a consensus appears to be forming that normal serum PCSK9 levels are somewhere around 100-1,000 ng/ml [47-53,57]. If these estimates for half-life and circulating levels are correct, then the liver must be constantly secreting large amounts of PCSK9 protein into the circulation. At one end of the spectrum, assuming a half-life of 30 minutes, a circulating level of 100 ng/ml and a plasma volume of 2.5 liters, the liver would be secreting 6 mg/day of PCSK9 protein into the plasma. At the other end of the spectrum, assuming a half-life of 5 min, a circulating level of 1,000 ng/ml and a plasma volume of 2.5 liters, the liver would be secreting 360 mg/day of PCSK9 protein into the plasma.

The true amount is probably somewhere between these 2 extreme estimates, but going through this exercise demonstrates that there is a significant amount of circulating PCSK9 protein that would have to be neutralized by a monoclonal antibody. As a result, it is far from certain that an anti-PCSK9 monoclonal antibody can be practically developed. The fact that it may be possible, however, combined with the fact that an anti-PCSK9 monoclonal antibody should theoretically be synergistic or at least additive to statins with regard to LDL-C lowering makes the development of such a biotherapeutic a clear goal.

If this goal cannot be attained, however, the data obtained so far showing that statins, fibrates and ezetimibe all increase serum PCSK9 levels strongly suggest that any novel LDL-C-lowering compound that acts to decrease PCSK9 levels should be able to be combined with the currently prescribed medications to result in further LDL-C lowering than has ever been possible to date. Furthermore, it may also be safe to speculate that novel lipid-lowering medications that are PCSK9 neutral (meaning that while they do not decrease, they also do not increase PCSK9 levels) may be combined with currently prescribed medications to enable greater LDL-C lowering than can be achieved at the present time.

## Conclusions

Certainly, the past decade has seen an explosion in our knowledge about PCSK9 and the role that it plays in the regulation of LDL-C levels. In less than 10 years, we have witnessed the discovery of the protein, the finding that mutations in PCSK9 can drastically affect LDL-C levels, an understanding of the mechanism by which PCSK9 functions, and the development of immunoassays to measure its circulating levels. These advances have allowed multiple researchers to measure PCSK9 protein levels in human serum and demonstrate that commonly prescribed lipid lowering medications (including statins, fibrates, and ezetimibe) increase circulating PCSK9 levels. Preliminary proof-of-concept studies showing that neutralizing anti-PCSK9 antibodies can significantly lower LDL-C have also occurred, suggesting that addition of PCSK9 inhibitors to current therapies may allow for lowering LDL-C to a greater extent than has previously been achievable. While it is impossible to predict accurately what the next decade will hold, if the past one is any indication, it is safe to bet that the development of novel LDL-C-lowering compounds that decrease or inhibit PCSK9 or that act through PCSK9-independent pathways will be a top priority of multiple research laboratories around the world.

## List of abbreviations

ASO: antisense oligonucleotide; HDL: high density lipoprotein; HDL-C: high density lipoprotein cholesterol; LDL: low density lipoprotein; LDLR: low density lipoprotein receptor; LDL-C: low density lipoprotein cholesterol; PCSK9: proprotein convertase subtilisin kexin type 9; TC: total cholesterol; TG: triglycerides; VLDL: very low density lipoprotein

## Competing interests

This work was supported entirely by Eli Lilly and Company.

## Authors' contributions

RK conceived of and wrote the manuscript. JT and GC helped to draft and critically edit the manuscript. All authors read and approved the final version of the manuscript.

## Authors' information

RK, JT, and GC are all employees of Eli Lilly and Company. RK is the Senior Director of the Laboratory for Experimental Medicine. JT is a scientist in the Laboratory for Experimental Medicine. GC is a Senior Research Advisor in the Cardiovascular/Metabolic Disease Drug Hunting Team.
